# Safety of SGLT-2 inhibitors in the management of heart failure in the adult congenital heart disease patient population

**DOI:** 10.1016/j.ijcchd.2024.100495

**Published:** 2024-02-17

**Authors:** Ahmed Kheiwa, Brian Ssembajjwe, Payush Chatta, Stephen Nageotte, Dmitry Abramov

**Affiliations:** a Division of Cardiology, Adult Congenital Heart Disease Program, Loma Linda University, Loma Linda, CA, USA; b Division of Internal Medicine, Department of Medicine, Loma Linda University, Loma Linda, CA, USA; c Division of Cardiology, Department of Medicine, Loma Linda University, Loma Linda, CA, USA; d Division of Pediatric Cardiology, Loma Linda University Children's Hospital, Loma Linda, CA, USA

## Abstract

**Background:**

Sodium glucose transporter 2 inhibitors (SGLT-2i) have shown safety and efficacy in patients with heart failure (HF). However, evidence for the use of SGLT-2i in adult congenital heart disease (ACHD) patients with HF is limited.

**Methods:**

We performed a retrospective, single center analysis of 18 patients (>18 years of age) with ACHD and a diagnosis of HF who were initiated on an SGLT-2i. Patient characteristics, including vital signs, laboratory values, concomitant medications, clinical outcomes, and echocardiograms, were obtained as part of standardized clinical care at our ACHD program before and 2–6 months after initiation of SGLT-2i. The primary outcome was to demonstrate safety of SGLT-2i initiation via potential changes in systolic blood pressure, serum sodium, and serum creatinine.

**Results:**

Of the 18 patients, 11 (61%) had moderate complexity congenital heart disease while 7 (39%) had great complexity congenital heart disease. Post initiation, there were no significant differences in systolic blood pressure (121.8 ± 20.8 mmHg to 114.4 ± 14.9 mmHg, p = 0.06), sodium level (138.7 ± 2.9 mMol/L to 138.0 ± 2.2 mMol/L, p = 0.75), and creatinine level (0.85 ± 0.18 mg/dL to 0.89 ± 0.18 mg/dL, p = 0.07). There was a statistically significant decline in weight (78.9 ± 22.9 kg to 76.0 ± 23.0 kg, p = 0.0039) but without a statistically significant change in NT-pro NBP (1358.2 ± 2735.0 pg/mL to 601.6 ± 786.1 pg/mL, p = 0.36).

**Conclusions:**

We demonstrated the use of SGLT-2i in a small cohort of ACHD population, including patients with complex congenital heart disease, appears safe and well tolerated. The safety and potential efficacy of SGLT-2i in patients with ACHD will require further evaluation in prospective multicenter studies.

## Highlights

• Use of SGLT-2i in ACHD patients, including patients with complex congenital heart disease, appears safe and well tolerated.

## 1 Introduction

The emergence of sodium-glucose cotransporter 2 inhibitors (SGLT-2i) as a pillar of guideline-directed medical therapy in heart failure (HF) management is due to its associated morbidity and mortality benefits [1]. SGLT-2i are cardioprotective through several mechanisms. SGLT-2i play an important role in blunting the sympathetic nervous system as competitive inhibitors of cardiac adrenergic receptors as well as by altering cardiac metabolism to be more ketone dependent [2,3]. In addition, It have been associated with reduced myofilament stiffness leading to improved diastolic parameters [4] such as reduced filling pressures. Clinical trials have shown that SGLT-2i as a class have been associated with improved outcomes in non-congenital HF patients with both reduced and preserved ejection fractions [1,5], as well as in other patient populations with diabetes or chronic kidney disease [6,7]. Although the benefits of SGLT-2i have been well demonstrated in the general adult HF population, evidence for use of these agents in adult congenital heart disease (ACHD) population is limited. We therefore sought to evaluate the safety of this therapy in a small cohort of ACHD patients.

## 2 Study design

This is a retrospective, single center analysis of patients >18 years of age with ACHD who were started on an SGLT-2i as part of routine clinical care at our institution. Consistent with guidelines for the general HF population, SGLT-2i were initiated in select patients with HF (functional class II, III, or IV) with reduced (≤40%), mildly reduced (40%–50%) or preserved (≥50%) ejection fractions. All patients were receiving appropriate management with other HF medications, appropriate surgical interventions, and device therapy for the management of HF and congenital heart disease in a dedicated clinic led by an ACHD-trained cardiologist. Patients were assessed at baseline (prior to initiation of SGLT-2i) and within a six-month follow up period; with patients typically seen 2–6 months post initiation of SGLT-2i for routine follow-up. Patient care, including follow-up visits, laboratory evaluation, and echocardiography were performed according to routine standard of care.

The primary safety endpoints included stable systolic blood pressure and renal function, including sodium and creatinine. Secondary endpoints included changes in weight, natriuretic peptides (NT-pro BNP), hematocrit, diuretic need, ejection fraction, and systemic ventricular volume. Data were obtained from chart review of the electronic medical record. Laboratory values and other data, before and after SGLT-2i initiation, were compared using two-sided paired T-Tests (Excel, Microsoft Corporation). The pre-SGLT-2i initiation laboratory data was compared to the respective lab values done within a 2–6 month period. This retrospective study was approved by the hospital Institutional Review Board and adheres to the institution's ethical standards.

## 3 Results

Between April 2022 and June 2023, we identified 18 ACHD patients who were initiated on an SGLT-2i, with all patients receiving dapagliflozin at 10 mg daily. The age ranged from 19 to 66 years old with an average of 37.0 ± 13.8 years. Eleven of the 18 patients (61%) had moderate complexity congenital heart disease while 7 (39%) patients had great complexity congenital heart disease (Table 1) as per 2018 AHA/ACC guideline for Adult with Congenital Heart Disease [8].

**Table 1:** Severity of adult congenital heart disease, organized as per the 2018 AHA/ACC guideline for Adults with Congenital Heart Disease.

Number of patients	Type of Adult Congenital Heart Disease
**Great Complexity ACHD**	
3 (17%)	2 Tricuspid atresia s/p Fontan palliation DORV, Mitral atresia s/p Fontan palliation
2 (11%)	Systemic RV (cc-TGA), Systemic RV (D- TGA s/p Mustard)
2 (11%)	Pulmonary atresia with VSD Pulmonary atresia With Intact Ventricular Septum (IVS)
**Moderate Complexity ACHD**	
1 (5%)	VSD/pulmonary stenosis
1 (5%)	CAVC
3 (17%)	ASD/cor triatriatum Dexter
1 (5%)	Ebstein anomaly
2 (11 %)	Tetralogy of Fallot
2 (11 %)	VSD, subaortic stenosis
1 (5%)	Congenital mitral valve dysplasia

**Abbreviations**: (AV): Atrioventricular, (VSD): Ventricular Septal Defect, (ASD): Atrial septal defect, (MAPCA): Makor aortopulmonary collateral arteries, (TGA): Transposition of Great Arteries, (DORV): Double outlet right ventricle, (PS): Pulmonic Stenosis, (SVC): Superior Vena Cava, (TAPVR): Total anomalous pulmonary venous return, (AR): Aortic regurgitation, (PDA): Patent ductus arteriosus.

Four out of 18 (22%) patients had single ventricle physiology; 3 patients post Fontan procedure and 1 with palliated single ventricle with Eisenmenger physiology. Seven patients had a pre-existing diagnosis of hypertension (39%), 4 (22%) had pre-diabetes, and 3 (17%) had diabetes. At baseline (prior to initiation of SGLT-2i), 9 (50%) patients had a systemic ventricular ejection fraction ≤40%, 5 (28%) had a mildly reduced ejection fraction (41–50%), and 4 (22%) had a preserved ejection fraction defined as an EF >50%. Characteristics of participants are included in Table 2.

**Table 2:** Display of patient characteristics for those with congenital heart disease. The racial group was reported by the patient. Body mass index (BMI) is the weight in kilograms divided by the square of the height in meters. Pre-diabetes was classified as a percent Hgb A1c value between 5.7 and 6.4 and diabetes was classified as a percent Hgb A1c value of >6.4.

Patient Characteristic	n (%)
**Age (Yrs)**	37.0 ± 13.8
0–30	6 (33%)
31–60	11 (62%)
>60	1 (5%)
**Race**	
Black	2 (11%)
Hispanic	6 (33%)
White	8 (44%)
Asian	2 (11%)
**Female Sex**	6 (33%)
**Body Mass Index**	
≤24.9	6 (33%)
25–29.9	7 (39%)
30–34.9	2 (11%)
≥35	3 (17%)
**Fontan Procedure**	3 (17%)
**Type of ACHD**	
Moderate Complexity	11 (61%)
Great Complexity	7 (39%)
**Hypertensive**	7 (39%)
**Diabetes**	
No	11 (61%)
Pre-Diabetes	4 (22%)
Diabetes	3 (17%)
**Systemic Ventricular EF**	
≤40%	9 (50%)
41–50%	5 (28%)
≥50%	4 (22%)

**Graph 1:**
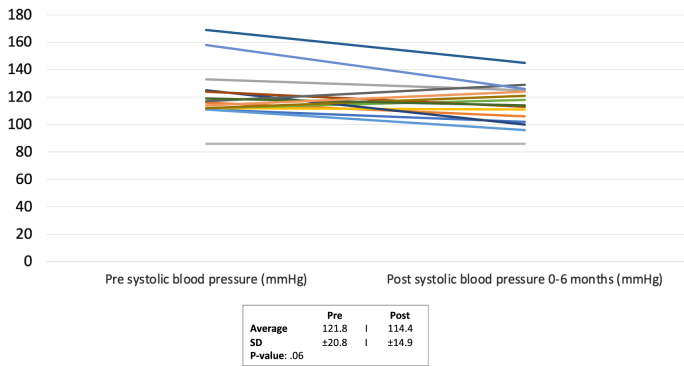
Blood pressure (mmHg) at baseline and during a 0–6 month follow up Pre and Post SGLT-2i Creatinine Levels.

Changes in primary and secondary endpoints, pre and post-SGLT-2i initiation, are show in Table 3. Baseline systolic blood pressure was 121.8 ± 20.8 mmHg and the subsequent systolic blood pressure post-initiation was 114.0 ± 14.9 mmHg (p = 0.06) (Graph 1). Of the 18 patients, 9 had a reduction of systolic blood pressure on their initial follow-up with the average reduction of 12 ± 9 mmHg and 4 had a reduction of SBP of >10 mmHg.

**Table 3:** Primary and secondary endpoints obtained at baseline (prior to initiation of SGLT2i) and during a 0–6 month follow up visit.

Primary Variable	Pre-SGLT-2I	Post SGLT-2I	*P*-Value
Systolic BP (mmHg)	122 ± 21	114 ± 15	0.06
Diastolic BP (mmHg)	77 ± 12	73 ± 9	
Creatinine (mg/dL)	0.85 ± 0.18	0.89 ± 0.18	0.07
GFR (ml/min/1.732)	105 ± 19	101 ± 21	0.06
Sodium (mMol/L)	138.7 ± 2.8	138 ± 2.2	0.75
Potassium (mMol/L)	4.1 ± 0.37	4.1 ± 0.36	0.83
Pro-BNP (pg/mL)	1358 ± 2735	602 ± 786	0.36
Hematocrit (%)	39.2 ± 6.7	40.1 ± 6.9	0.22
Weight (kg)	78.9 ± 22.9	76.0 ± 23.0	0.0039

## 4 Laboratory values

**Graph 2:**
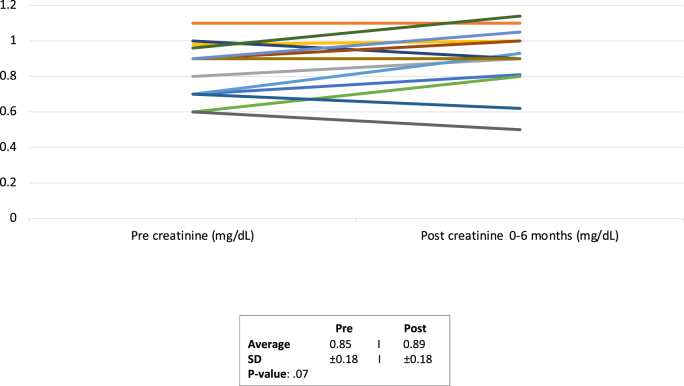
Creatinine value (mg/dL) at baseline and during a 0–6 month follow up Pre and Post SGLT-2i Weight changes.

**Graph 3:**
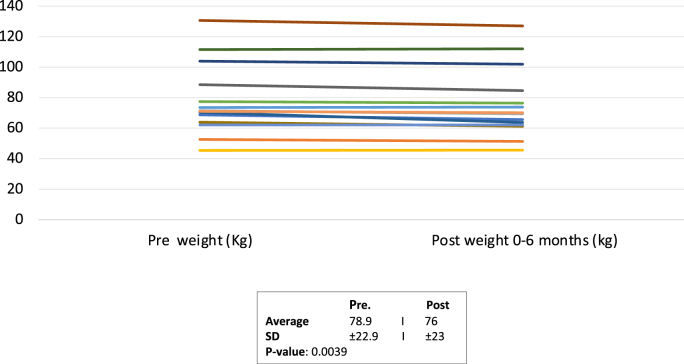
Change in weight (kg) at baseline and during a 0–6 month follow up.

The average creatinine value was 0.85 ± 0.18 mg/dL at baseline and 0.89 ± 0.18 mg/dL (p = 0.07) on follow up (Graph 2). The estimated glomerular filtration rate (eGFR) was calculated via the Chronic Kidney Disease Epidemiology 2020 equation and resulted with an average value of 105.0 ± 19.1 mL/min/1.73^2^ at baseline and 100.0 ± 21.2 mL/min/1.73^2^ at follow up (p = 0.06). The average sodium level was 138.7 ± 2.8 mMol/L at baseline and 138.0 ± 2.2 mMol/L at follow up (p = 0.75) and potassium level was 4.1 ± 0.37 mMol/L at baseline and 4.1 ± 0.36 mMol/L at follow up (p = 0.83). Average baseline hematocrit was 39.2 ± 6.7% and 40.1 ± 6.9% at follow up (p = 0.22). There was a statistically significant decline in weight (78.9 ± 22.9 kg to 76.0 ± 23.0 kg, p = 0.0039) (Graph 3). Of the study population, 5/18 (27%) had a NT-pro BNP done within six months of initiation, with the average value at baseline being 1358 ± 2735 pg/mL and 601 ± 786.1 pg/mL at follow up (p = 0.36). Of those with an available serial NT-pro BNP, 4 patients (80%) demonstrated a decrease, and 1 patient (20%) demonstrated an increase in their NT-pro BNP

.

Nine patients had a repeat echocardiogram (within a six month follow up) to assess changes in systemic ventricular (EF). Of the 9 patients, 1 initially had a greater than 10% reduction in ejection fraction (decreased from 45-50% to 30–35%), which later improved to 50–55%; 4 patients ejection fraction remained the same, and another 4 had an improvement in EF. Among the 4 patients with improvement in systemic EF, the improvements were of 35–40%, 32–45%, 15–65%, and 25–40%.

Out of the 18 patients, 13 (72%) were on a loop diuretic (furosemide) prior to initiation of a SGLT-2i. Total daily dosing ranged from 20 to 80 mg daily. Within a six month follow up period, 14 (78%) had no change in the diuretic regimen, 2 (11%) had an increase in the amount of scheduled diuresis, and 2 (11%) patients had a reduction of diuretic dose. NYHA classification was also assessed to evaluate functional status as part of routine care during clinic visits. At baseline, 13 were NYHA class II, 4 patients were NYHA class III, and 1 patient had NYHA class IV symptoms. Seventeen patients had clinic follow up within six months with the following NYHA classification of functional status: 16 with NYHA class II, and 1 with NYHA class III. Of these 17 patients, 13 had no significant change in functional status and 4 had an overall improvement in their functional capacity as determined by the NYHA classification. There were no other significant side effects noted from SGLT-2i initiation in this cohort, including urinary tract infections or genital fungal infections during the 6-month follow-up period.

## 5 Discussion

This retrospective analysis describes the use of SGLT-2i in 18 ACHD patients with HF, including in patients with complex congenital heart disease and single ventricle physiology. We found that initiation of SGLT2i was well tolerated during short term follow up. Specifically, there was no significant worsening of renal function or reduction in blood pressure after initiation of SGLT-2i in a heterogenous group of ACHD patients. Our results add to the literature on the use of SGLT-2i in patients with ACHD. To the best of our knowledge, this is the first study to evaluate SGLT-2i in a complex ACHD cohort that included patients with single ventricle circulation.

There is a scarcity of data regarding the use of SGLT-2i in the ACHD population. In a small cohort of 10 patients with systemic RV failure, dapagliflozin was well-tolerated with an initial slight decline of eGFR that later recovered to baseline at 6 month follow up [9]. In a slightly larger study including 35 ACHD patients, (including 8 patients with systemic RV failure), SGLT-2i was also well tolerated with no significant worsening of renal function [10]. Our study, in line with these findings, provides further data for the safety of dapagliflozin in ACHD population with no significant worsening of renal function or change in blood pressure at short term follow up. The cohort in our study is more heterogenous and includes patients with single ventricle physiology which was not included in the previous two studies.

Heart failure is one of the most challenging clinical dilemmas in the ACHD population, causing a significant impact on morbidity and mortality. It has been recognized as one of the leading causes of death in the ACHD population and has led to a significant increase in hospital admissions over the past decade [11]. Heart failure is more common among patients with systemic right ventricle or with single ventricle physiology, and it is therefore important to include a heterogenous cohort of ACHD patients in studies of novel cardiovascular drugs. Compared to patients with acquired heart disease who develop HF, ACHD patients often present with unique HF phenotypes. It is commonly caused by heterogenous anatomical and hemodynamic derangements including shunt lesions with volume or pressure overload, obstructive lesions as in coarctation of aorta, systemic right ventricular failure, single ventricle failure, or chronic cyanosis. The two most unique phenotypes are patients with systemic RV dysfunction and single ventricle dysfunction [12].

The process of neurohormonal overactivation has been well demonstrated in the non-congenital HF population and correlates with worsening ventricular function and disease severity [13]. Modifying the neurohormonal overactivation through standard HF therapy including ACEIs/ARB/angiotensin receptor-neprilysin inhibitor (ARNi), beta blockers, mineralocorticoid antagonists (MRA), and SGLT-2i has become the pillar of guideline directed medical therapy for HF patients in the non-congenital population. Regarding the pathophysiology of HF in the ACHD population, neurohormonal overactivation has similarly been shown to correlate with disease severity and ventricular dysfunction [11]. The process includes activation of the sympatho-adrenergic system, renin-angiotensin-aldosterone system, and endothelin system leading to vasoconstriction, sodium retention, and maladaptive ventricular remodeling [14]. It seems intuitive then to speculate that pharmacological manipulation of the neurohormonal pathways in ACHD patients may provide similar benefit along the same line of applying pharmacological therapy in non-congenital HF patients. However, the data reported in that regard for other agents have not been supportive of this hypothesis. In a randomized controlled trial including 88 symptomatic patients with systemic RV failure (including CC-TGA and D-TGA post Mustard palliation), valsartan showed no significant benefit with no significant improvement in Right Ventricular systolic function or exercise capacity [15]. In another report, ARNI demonstrated an improvement in NT -proBNP level in patients with systemic RV failure, however it didn't reveal significant improvement in ventricular function or exercise capacity [16]. High rates of sinus node dysfunction and bradyarrhythmias limit the routine use of high dose of beta blocker therapy in this population. Given the limited data on the use of SGLT-2i in this patient population, our focus was to evaluate the safety of this fundamental HF therapy in ACHD population.

The use of SGLT2i comes with a risk profile of its own. As shown in the landmark trial EMPEROR-Preserved, uncomplicated genital or urinary tract infections and hypotension were two adverse events encountered in patients being treated with SGLT-2i [1]. Furthermore, an initial decline in eGFR following initiation of SGLT2i [7,17] and reduction in systolic blood pressure have been reported [18]. Keeping these adverse effects in mind, the primary goal of our study was to assess safety in ACHD patients with HF. Although longer term studies will be needed to better characterize risk, our results did not show a significant change in either renal function or systolic blood pressure post SGLT2i initiation on our population. Hemoconcentration due to osmotic diuresis and natriuresis have been reported in patients treated with SGLT-2i [19]. Our study did not demonstrate a significant change in hematocrit over the follow up period although this may be due to the small sample size of our cohort. Additionally, several studies have demonstrated a statistically significant weight loss, that is attributed to loss of plasma volume with increased duiresis of extracellular volume, reduction in adipose tissue and daily urine negative calorie loss [20]. Our study was in line with prior studies, in showing a statistically significant reduction in weight, which warrants further evaluation of the significance and the type of weight loss achieved.

Safety outcomes regarding initiation of SGLT-2i and renal function were studied as renal function plays an imperative role in HF exacerbations. While SGLT-2i have shown renal protection, some studies have shown an initial decline in eGFR after initiation of a SGLT-2i [7]. The initial reduction may result in early discontinuation of SGLT-2i. However, it should be noted that the observed decline in eGFR was observed in patients with type 2 diabetes [13,14]. This could be attributed to differences in renal pathology in those with type two diabetes versus heart failure. Such deleterious effects of SGLT-2i on renal function has been refuted by Adamson et al. who demonstrated that, in patients randomized to dapagliflozin, those with an initial reduction in eGFR had better outcomes than those who did not, without safety concerns [7]. This initial drop in eGFR was not observed in our study, although this may be due to small sample size. Additionally, electrolytes including sodium and potassium, did not have a significant change either.

As a pillar of treatment in patients with heart failure, studies demonstrating the safety/efficacy profile of SGLT-2i in patients with CHD are lacking. Our study is unique in that it evaluates the safety of SGLT-2i in patients with CHD ranging from moderate to great complexity, including those with single ventricle physiology and systemic right ventricular failure. To our knowledge, we report one of the largest case series in patients with such complexity. In this study, we demonstrate that initiation of SGLT-2i in patients with ACHD, including those with single ventricle and systemic right ventricle, is feasible and safe. Initiation was well tolerated without significant adverse effects on hemodynamic parameters including blood pressure and renal function. Initiation in some patients was associated with weight loss, reduction in NT-pro BNP, as well as an improvement in NYHA class, although our small and non-randomized analysis is not sufficient to assign causation to these changes. These findings will require further evaluation in large scale prospective multicenter studies and expand on the limited prior data of the use of SGLT-2i in patients with ACHD.

## 6 Limitations

This was a single center non-randomized cohort with a small sample size and data was retrospectively analyzed. Additionally, practice variability between ACHD centers may not be generalizable to all other centers. However, our data expands on prior studies demonstrating safety of SGLT2i in a number of ACHD patient populations, which may provide further reassurance about this medication class and facilitate future prospective studies. The lack of significant side effects, additional potential benefits, or laboratory derangements may be due to the small sample size and short duration of follow up. The data included only includes follow-up and studies performed as part of standard of care.

## 7 Conclusion

In conclusion, we report that the use of SGLT-2i in our small cohort of patients with heterogenous ACHD appears safe and well tolerated. The safety and potential efficacy of SGLT-2i in patients with ACHD will require further evaluation in large scale prospective multicenter studies.

## CRediT authorship contribution statement

**Ahmed Kheiwa:** Writing – review & editing, Supervision, Formal analysis, Data curation, Conceptualization. **Brian Ssembajjwe:** Writing – review & editing, Writing – original draft, Data curation. **Payush Chatta:** Writing – review & editing, Writing – original draft, Data curation. **Stephen Nageotte:** Writing – review & editing. **Dmitry Abramov:** Writing – review & editing, Supervision, Formal analysis, Data curation, Conceptualization.

## Declaration of competing interest

The authors declare that they have no known competing financial interests or personal relationships that could have appeared to influence the work reported in this paper.
